# Discovering biomarkers for antidepressant response: protocol from the Canadian biomarker integration network in depression (CAN-BIND) and clinical characteristics of the first patient cohort

**DOI:** 10.1186/s12888-016-0785-x

**Published:** 2016-04-16

**Authors:** Raymond W. Lam, Roumen Milev, Susan Rotzinger, Ana C. Andreazza, Pierre Blier, Colleen Brenner, Zafiris J. Daskalakis, Moyez Dharsee, Jonathan Downar, Kenneth R. Evans, Faranak Farzan, Jane A. Foster, Benicio N. Frey, Joseph Geraci, Peter Giacobbe, Harriet E. Feilotter, Geoffrey B. Hall, Kate L. Harkness, Stefanie Hassel, Zahinoor Ismail, Francesco Leri, Mario Liotti, Glenda M. MacQueen, Mary Pat McAndrews, Luciano Minuzzi, Daniel J. Müller, Sagar V. Parikh, Franca M. Placenza, Lena C. Quilty, Arun V. Ravindran, Tim V. Salomons, Claudio N. Soares, Stephen C. Strother, Gustavo Turecki, Anthony L. Vaccarino, Fidel Vila-Rodriguez, Sidney H. Kennedy, on behalf of the CAN-BIND Investigator Team

**Affiliations:** University of British Columbia and Vancouver Coastal Health Authority, 2255 Wesbrook Mall, Vancouver, BC V6T 2A1 Canada; Queen’s University, Providence Care, Mental Health Services 752 King Street West, Postal Bag 603, Kingston, ON K7L 7X3 Canada; University Health Network, 399 Bathurst Street, Toronto, ON M5T 2S8 Canada; Department of Psychiatry, University of Toronto, 250 College Street, 8th floor, Toronto, ON M5T 1R8 Canada; Centre for Addiction and Mental Health, 1001 Queen St. W, Toronto, ON M6J 1A8 Canada; University of Ottawa Institute of Mental Health Research, 1145 Carling Avenue, Ottawa, ON K1Z 7K4 Canada; Loma Linda University, 24851 Circle Dr, Loma Linda, CA 92354 USA; Indoc Research, 258 Adelaide St. East, Suite 200, Toronto, ON M5A 1N1 Canada; Department of Pathology and Molecular Medicine, Queen’s University, 88 Stuart Street, Kingston, ON K7L 3N6 Canada; McMaster University, and St. Joseph’s Healthcare Hamilton, 1280 Main Street West, Hamilton, ON L8S4L8 Canada; Department of Psychology, Queen’s University, Kingston, ON K7L 3N6 Canada; Aston University, Aston Triangle, Birmingham, West Midlands B4 7ET UK; University of Calgary Hotchkiss Brain Institute, 2500 University Dr NW, Calgary, AB T2N 1N4 Canada; University of Guelph, 50 Stone Rd E, Guelph, ON N1G 2W1 Canada; Simon Fraser University, 8888 University Dr, Burnaby, BC V5A 1S6 Canada; Universisty of Michigan, 500S State St, Ann Arbor, MI 48109, USA; University of Reading, Earley Gate, Whiteknights, Reading, RG6 6AL UK; St. Michael’s Hospital, 193 Yonge St, Toronto, ON M5B 1M4 Canada; Rotman Research Institute at Baycrest Centre, 3560 Bathurst Street, Toronto, ON M6A 2E1 Canada; McGill University , 845 Rue Sherbrooke O, Montréal, QC H3A 0G4 Canada; Douglas Mental Health University Institute Frank B. Common (FBC) F-3145, 6875 LaSalle Boulevard, Montréal, QC H4H 1R3 Canada

## Abstract

**Background:**

Major Depressive Disorder (MDD) is among the most prevalent and disabling medical conditions worldwide. Identification of clinical and biological markers (“biomarkers”) of treatment response could personalize clinical decisions and lead to better outcomes. This paper describes the aims, design, and methods of a discovery study of biomarkers in antidepressant treatment response, conducted by the Canadian Biomarker Integration Network in Depression (CAN-BIND). The CAN-BIND research program investigates and identifies biomarkers that help to predict outcomes in patients with MDD treated with antidepressant medication. The primary objective of this initial study (known as CAN-BIND-1) is to identify individual and integrated neuroimaging, electrophysiological, molecular, and clinical predictors of response to sequential antidepressant monotherapy and adjunctive therapy in MDD.

**Methods:**

CAN-BIND-1 is a multisite initiative involving 6 academic health centres working collaboratively with other universities and research centres. In the 16-week protocol, patients with MDD are treated with a first-line antidepressant (escitalopram 10–20 mg/d) that, if clinically warranted after eight weeks, is augmented with an evidence-based, add-on medication (aripiprazole 2–10 mg/d). Comprehensive datasets are obtained using clinical rating scales; behavioural, dimensional, and functioning/quality of life measures; neurocognitive testing; genomic, genetic, and proteomic profiling from blood samples; combined structural and functional magnetic resonance imaging; and electroencephalography. De-identified data from all sites are aggregated within a secure neuroinformatics platform for data integration, management, storage, and analyses. Statistical analyses will include multivariate and machine-learning techniques to identify predictors, moderators, and mediators of treatment response.

**Discussion:**

From June 2013 to February 2015, a cohort of 134 participants (85 outpatients with MDD and 49 healthy participants) has been evaluated at baseline. The clinical characteristics of this cohort are similar to other studies of MDD. Recruitment at all sites is ongoing to a target sample of 290 participants. CAN-BIND will identify biomarkers of treatment response in MDD through extensive clinical, molecular, and imaging assessments, in order to improve treatment practice and clinical outcomes. It will also create an innovative, robust platform and database for future research.

**Trial registration:**

ClinicalTrials.gov identifier NCT01655706. Registered July 27, 2012.

## Background

Depressive disorders, including Major Depressive Disorder (MDD), are highly prevalent and disabling conditions with substantial personal and societal costs [1]. MDD is now the second leading cause of disability worldwide [2] and contributes to excess mortality associated with many comorbid medical conditions [3]. Treatment of depressive disorders is based on empirical data and evidence-based guidelines, but treatment selection remains more of an art than a science. Hence, the discovery of clinical and biological markers, or biomarkers, of treatment response that would inform an individualized approach to depression treatment remains a research goal [4].

A major challenge to identify predictors (baseline characteristics that predict response), moderators (baseline characteristics that predict differential response to a specific treatment) and mediators (events or changes occurring during treatment that explains the response) is that MDD is a complex, heterogeneous condition. A variety of neurobiological and environmental influences, both independently and in combination with one another, can alter the clinical expression of MDD in terms of symptoms, severity, episode duration, response to treatment, and functional outcomes. As a result, no single intervention is effective for all people with depression. Current diagnostic systems such as the DSM-5 [5] can reliably codify depressive symptoms as criteria for MDD, but these symptoms are not unique to depression and, even if clustered together, may not represent a specific underlying disease process or a treatment substrate. Hence, the clinical entity termed “Major Depressive Disorder” represents only the final, external manifestations of an enormously complex, multi-level, multi-factorial process.

The Canadian Biomarker Integration Network in Depression (CAN-BIND) [6] was created with an aim to use an integrated approach to biomarker discovery. CAN-BIND draws on multidisciplinary expertise from investigative teams at 8 Canadian universities, all in active collaboration with the Ontario Brain Institute (OBI) [7] and Indoc Research (Toronto, ON, Canada). The Canadian Institutes of Health Research, academic institutions, and various industry partners provide additional funding and support (see Acknowledgements).

The overall goal of CAN-BIND is to identify predictors, moderators, and mediators of treatment response and non-response in people with MDD to guide clinical decision-making. CAN-BIND-1 uses an integrated clinical, neuroimaging and molecular approach with high-dimensional mathematical modeling techniques to specifically search for (a) baseline predictors and moderators of antidepressant and adjunctive agent response, (b) early treatment mediators of response (changes from baseline to 2 weeks), and (c) later treatment mediators of response (changes from baseline to 8 weeks, and to 16 weeks).

## Methods

### Overview of protocol

Patients with MDD are treated with open-label escitalopram 10–20 mg/d for 8 weeks. Responders (≥50 % reduction in Montgomery-Åsberg Depression Rating Scale [MADRS] score) continue on escitalopram for another 8 weeks, while non-responders have aripiprazole 2–10 mg/d added on to escitalopram for 8 weeks. Clinical, neuroimaging and molecular assessments are conducted at Baseline (Week 0) and Weeks 2 and 8; clinical and molecular assessments also are conducted at Weeks 4, 10, and 16; and additional brief clinical evaluations are completed at Weeks 6, 12, and 14. Clinical characterization assesses a broad palette of symptoms, functional outcomes, cognitive performance, personality dimensions, and recent and past life events.

### Participants

Participants are recruited at 6 clinical centres: Vancouver (Djavad Mowafaghian Centre for Brain Health), Calgary (Hotchkiss Brain Institute), Toronto (2 sites: University Health Network and Centre for Addiction and Mental Health), Hamilton (St. Joseph’s Healthcare Hamilton), and Kingston (Providence Care, Mental Health Services). Research Ethics Boards at each site approved the study. Recruitment draws upon outpatient-referral networks, community-based advertising, and dedicated knowledge translation (KT) activities.

Table 1 lists the inclusion and exclusion criteria for the patients with MDD. Healthy comparison participants are 18–60 years of age, with no psychiatric or unstable medical diagnosis, and sufficient fluency in English to complete study procedures. They are matched to the patient group by sex and age distribution.

**Table 1:** Inclusion and exclusion criteria for patients

*Inclusion Criteria*	
• Outpatients 18 to 60 years of age.	
• DSM-IV-TR criteria for MDE in MDD, as confirmed by the MINI.	
• Depressive episode duration ≥3 months.	
• Free of psychotropic medications for at least 5 half-lives (i.e. 1 week for most antidepressants, 5 weeks for fluoxetine) before baseline.	
• Score ≥24 on the MADRS.	
• Fluent in English, sufficient to complete the interviews and self-report questionnaires.	
*Exclusion Criteria*	
• Diagnosis of Bipolar I or Bipolar II disorder.	
• Any other psychiatric diagnosis that is considered the primary diagnosis.	
• Any significant personality disorder diagnosis (e.g., borderline, antisocial) that might interfere with participation in the protocol, defined by clinician judgment.	
• High suicidal risk, defined by clinician judgment.	
• Substance dependence/abuse in the past 6 months.	
• Significant neurological disorders, head trauma, or other unstable medical conditions.	
• Pregnant or breastfeeding.	
• Psychosis in the current episode.	
• High risk for hypomanic switch (i.e., history of antidepressant-induced hypomania).	
• Failed 4 or more adequate pharmacologic interventions (as determined by the ATHF).	
• Previously failed or showed intolerance to escitalopram or aripiprazole.	
• Started psychological treatment within the past 3 months with the intent of continuing treatment.	
• Contraindications to MRI.	

DSM-IV-TR, Diagnostic and Statistical Manual of Mental Disorders, Fourth Edition, Text Revision; MDE, Major Depressive Episode; MDD, Major Depressive Disorder; MINI, Mini International Neuropsychiatric Interview; MADRS, Montgomery-Åsberg Depression Rating Scale; ATHF, Antidepressant Treatment History Form

### Procedure

At the Screening Visit, eligible participants provide written, informed consent for all study procedures. Each patient undergoes screening evaluations that include a full psychiatric consultation to confirm a diagnosis of MDD using the Mini International Neuropsychiatric Interview (MINI) [8]. Previous medication history (type, dose, and duration) is collected using the Antidepressant Treatment History Form (ATHF) [9]. A detailed medical history and listing of concomitant treatments, if any, are recorded. A reproductive/menstrual history is obtained from female patients. Patients undergo medical work-up that includes physical examination, height and body weight measurements, clinical laboratory assays, and 12-lead electrocardiography (if indicated). Social background is collected using standardized reporting forms for demographic characteristics, handedness, ethnicity, education (expressed as years of formal schooling), marital status, occupational status, job classification, and household income. Participants are screened for contraindications to magnetic resonance imaging (MRI).

**Fig. 1:**
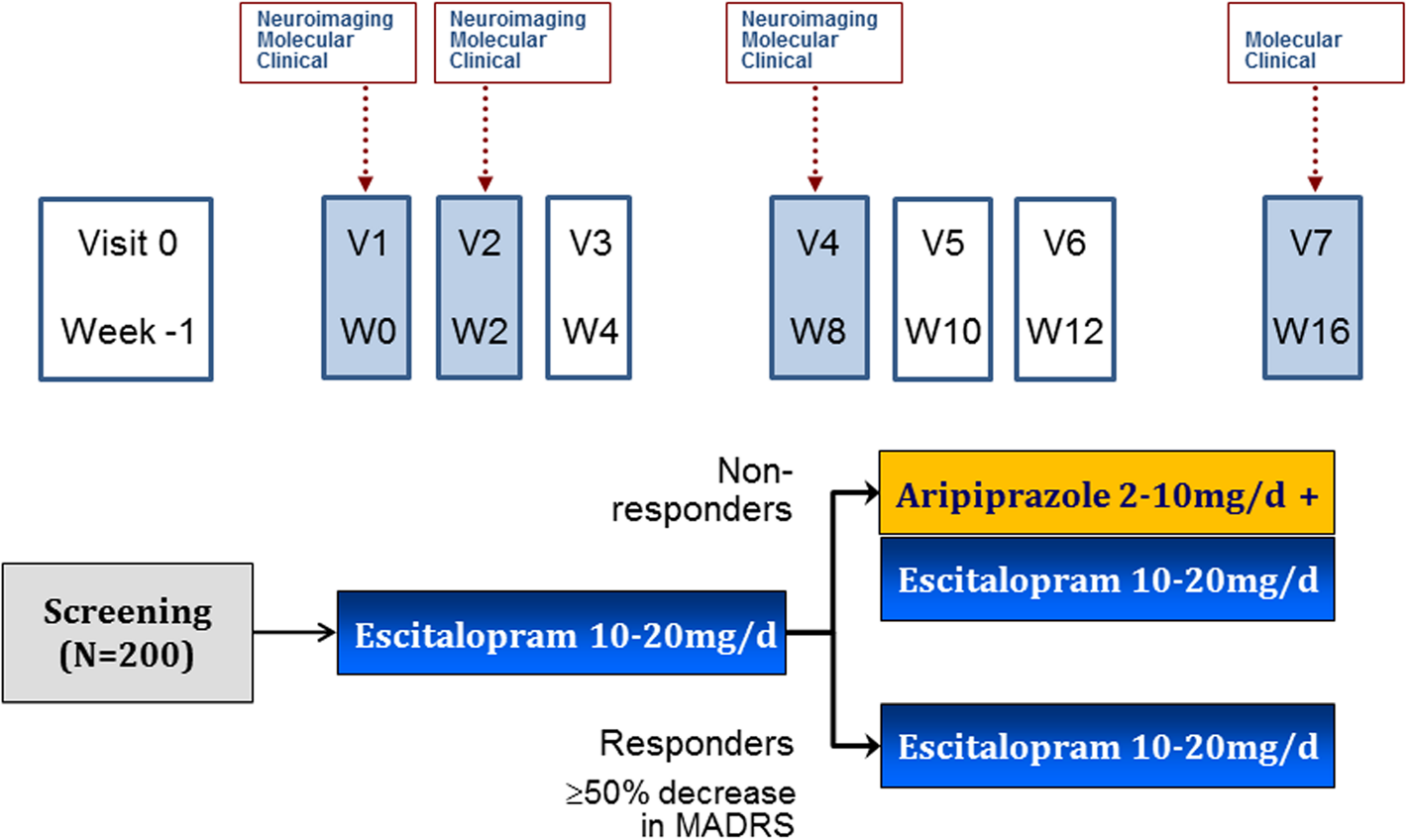
CAN-BIND-1 Clinical Protocol

Figure 1 shows an overview of the protocol. At the Baseline Visit (Visit 1, Week 0), extensive clinical assessments are conducted, and blood and urine samples are obtained for molecular analysis. Participants also undergo the first of 3 sessions of structural and functional neuroimaging and electroencephalography (EEG), as described below. All patients start treatment with escitalopram and receive standardized clinical management based on the CANMAT clinical guidelines [10].

Neuroimaging/EEG, molecular and clinical assessments are conducted again at Weeks 2 and 8. Additional blood samples are collected at Week 4 for pharmacogenetic analysis, and at Weeks 2, 10, and 16 for medication levels. Blood chemistry screening, urinalysis, and body weight measurements are repeated at Week 16. At the conclusion of the study, patients are discharged into standard clinical care by a family physician and/or regular psychiatrist. Patients can also elect to enrol in a long-term naturalistic follow-up study that includes wellness monitoring using electronic mental health (e-Mental Health) tools.

Healthy comparison participants attend 5 study visits: Screening, Baseline, Week 2, Week 8, and Week 16. They complete the same assessments as patients but do not receive any treatment.

### Treatments

In Phase 1, patients receive flexibly dosed escitalopram (10–20 mg/d) for 8 weeks. Patients are started at 10 mg/d and increased to 20 mg/d at Week 2 if they do not achieve ≥20 % reduction in MADRS score from baseline, and at Week 4 for those who do not achieve ≥50 % reduction in MADRS.

In Phase 2, patients who achieve ≥50 % reduction in baseline MADRS score at Week 8 are considered “responders” and continue on their effective dose of escitalopram for a further 8 weeks. Patients who do not achieve ≥50 % reduction in MADRS are considered “non-responders” and receive flexibly dosed aripiprazole (2–10 mg/d), added to escitalopram, for a further 8 weeks. Dose increases of aripiprazole are recommended if patients do not achieve ≥50 % reduction in MADRS after 2 or 4 weeks.

This standardized algorithm reflects usual clinical practice, is consistent with evidence-based treatment guidelines [11–13], and promotes consistency across all sites. Doses can be decreased at the discretion of the treating psychiatrist if patients do not tolerate higher doses. Treatment is open-label, and no randomization procedures are used. Medication adherence is monitored with pill counts at each visit.

Concomitant non-psychotropic medications for stable conditions are allowed at the discretion of the study psychiatrist, who can also permit use of vitamins, supplements, oral contraceptives, and non-prescription analgesics. Patients on pre-existing stable doses are allowed to continue on zopiclone up to 7.5 mg prn or lorazepam 1–2 mg prn, to a maximum of 3 doses/week.

### Assessments

#### Clinical platform

The clinical assessments (Table 2) are selected based on theoretical and clinical utility and to minimize respondent burden as much as possible. Raters received training, and inter-rater reliability was established using recorded interviews. Clinician-rated symptom and clinical measures include the Montgomery-Asberg Depression Rating Scale (MADRS), using the structured interview guide (SIGMA) to enhance reliability [14, 15], and Clinical Global Impression, Severity and Improvement scales (CGI-S and CGI-I) [16]. Depressive and other associated symptoms are explored in greater detail using the Depression Inventory Development (DID) semi-structured interview, as part of the International Society for CNS Drug Development’s initiative to refine and validate a new measurement tool for use in clinical trials of MDD [17]. Patient-rated symptom scales include the Quick Inventory for Depressive Symptomatology (QIDS-SR) [18] and the Generalized Anxiety Disorder (GAD-7) scale [19]. Manic and hypomanic symptoms are assessed with the clinician-rated Young Mania Rating Scale (YMRS) [20] and the patient-rated Hypomania Check-List (HCL-32) [21].

**Table 2:** Clinical characterization assessments

Clinician-Administered Assessments
Montgomery Asberg Depression Rating Scale (MADRS)
Young Mania Rating Scale (YMRS)
Clinical Global Impression (CGI)
Depression Inventory Development (DID) Interview
Toronto Side Effects Scale (TSES)
Sexual Side Effects Questionnaire (SexFX)
Childhood Experience of Care and Abuse (CECA)
Life Events and Difficulties Schedule (LEDS)
CNS Vital Signs (CNS-VS) computerized neuropsychological test battery
National Adult Reading Test (NART)
Self-Report Assessments
Quick Inventory of Depressive Symptomatology, Self-Report (QIDS-SR)
Generalized Anxiety Disorder 7-item scale (GAD-7)
Hypomania Check-List (HCL-32)
Brief Pain Inventory (BPI)
Sheehan Disability Scale (SDS)
Lam Employment Absence and Productivity Scale (LEAPS)
Quality of Life, Enjoyment and Satisfaction Questionnaire (Q-LES-Q)
World Health Organization Quality of Life Assessment (WHOQoL-BREF)
Pittsburgh Sleep Quality Index (PSQI)
Seasonal Pattern Assessment Questionnaire (SPAQ)
Biological Rhythm Interview of Assessment in Neuropsychiatry (BRIAN)
Dimensional Anhedonia Rating Scale (DARS)
Snaith-Hamilton Pleasure Scale (SHAPS)
Behavioural Inhibition/Behavioural Activation System (BIS/BAS)
NEO Five-Factor Inventory (NEO-FFI)
Experiences in Close Relationships (ECR-R) questionnaire
List of Threatening Experiences (LTE)
International Physical Activity Questionnaire (IPAQ)
Brief Diet Questionnaire

Self-rated scales are used to assess functional impairment (Sheehan Disability Scale; SDS) [22] and occupational functioning (Lam Employment Absence and Productivity Scale; LEAPS) [23]. Quality of life is measured both generally (World Health Organization Quality of Life Assessment; WHOQoL-BREF) [24] and with a specific focus on depression (Quality of Life, Enjoyment and Satisfaction Questionnaire; Q-LES-Q) [25], using these 2 self-rated measures.

We also assessed other behavioural and dimensional constructs, including aversive and incentive motivation (Behavioural Inhibition System/Behavioural Activation System; BIS/BAS) [26], anhedonia (Dimensional Anhedonia Rating Scale; DARS; and Snaith-Hamilton Pleasure Scale; SHAPS) [27, 28], personality (NEO Five-Factor Inventory; NEO-FFI) [29] and pain (Brief Pain Inventory; BPI-SF) [30]. Sleep, circadian rhythms, and seasonality are assessed using the Pittsburgh Sleep Quality Index (PSQI) [31], the Biological Rhythms Interview of Assessment in Neuropsychiatry (BRIAN) [32], and the Seasonal Pattern Assessment Questionnaire (SPAQ) [33]. Physical activity is measured using the self-rated International Physical Activity Questionnaire (IPAQ) [34], while dietary habits are assessed through a series of brief questions.

Assessment of environmental stressors includes adult patterns of attachment and recent stressful life events that are captured with self-report scales, including the Experience in Close Relationships (ECR-R) [35] scale, and the List of Threatening Experiences (LTE) [36], respectively.

Two clinical interviews are conducted by centralized, trained raters at Queen’s University and facilitated by Medeo, a secure telehealth/videohealth service (Medeo, Vancouver, BC, Canada). The Childhood Experience of Care and Abuse (CECA) [37], administered at Week 4 to patients and at Week 2 to healthy participants, is a semi-structured interview that carefully assesses childhood maltreatment. The Life Events and Difficulties Schedule (LEDS) [38], administered at Week 16 to all participants, is a semi-structured contextual interview and rating system that assesses stressful life events that have occurred within 6 months of depression onset. Interviews are audiotaped and transcribed so that responses can be coded based on standard, manualized conventions.

CNS Vital Signs (CNS VS) is a computerized test battery used to assess memory, reaction time and psychomotor speed, complex attention, and cognitive flexibility [39]. The reliability of CNS VS is similar to that of conventional neuropsychological tests [40]. The CNS VS has a robust normative database and is sensitive to common causes of cognitive impairment. Analyses of cognitive data will be augmented with an estimate of premorbid intelligence obtained using the National Adult Reading Test (NART) [41].

Medication side effects are documented with the clinician-rated Toronto Side Effects Scale (TSES) [42], which records the incidence, frequency, and severity of common adverse events, and the Sexual Functioning Questionnaire (SexFX), which evaluates specific sexual functioning [43]. Adverse Events are classified as mild, moderate or severe.

#### Neuroimaging platform

##### fMRI

Structural and functional neuroimaging data are obtained on 3.0 Tesla (3 T) magnetic resonance imaging (MRI) systems using multicoll phased-array head coils. Among the 6 clinical sites, 4 different models of scanners are used, thus mandating an extensive and ongoing standardization and quality control process to ensure that data are comparable and usable [44]. The four models of scanners include Discovery MR750 3.0T (GE Healthcare, Little Chalfont, Buckinghamshire, UK), Signa HDxt 3.0T (GE Healthcare, Little Chalfont, Buckinghamshire, UK), MAGNETOM TrioTim (Siemens Healthcare, Erlangen, Germany), and Achieva 3.0T (Philips Healthcare, Best, Netherlands). Cross-site T1 piloting included a “human phantom”, who travelled to each site for anatomical scans, and a manganese chloride (MnCl_2_)-based phantom model for progressive quantitative assessment of hydration based on signal intensity linearity characteristics [45]. Since the study’s launch, each site has obtained monthly scans of 2 geometric phantoms (a spherical agar phantom developed by the Function Bioinformatics Research Network, and a custom-built cylindrical model using plastic LEGO blocks) [46, 47] to facilitate scanner calibration and troubleshooting over the long term.

Following surveys and localization, the examination protocol includes a whole-brain T1-weighted turbo gradient echo sequence (9 min) at 1 mm^3^ resolution, repetition time (TR) = 6.2–1900 ms, echo time (TE) = 2.7–3.5 ms, flip angle = 8–15°, inversion time (TI) = 0–1100 ms, field of view (FOV) = 220–256 mm, matrix 256^2^–512^2^, 170–180 contiguous slices at 1 mm thickness. A small Vitamin E capsule is placed as a stereotactic marker at the right temple to confirm subject orientation during image review [48]. Secondly, a whole-brain diffusion tensor imaging (DTI) series is obtained using 30 gradient directions at 2 b-values (500 and 1000 s/mm^2^) (5 min) with an additional 3 images at b = 0 s/mm^2^ for tensor construction at a final voxel resolution of 2.3 x 2.3 x 5 mm (1 min). Functional MRI includes a 10-min resting state scan with eyes open using a fixation cross [49], obtained using a whole-brain T2*-sensitive blood oxygenation level-dependent (BOLD) echo planar imaging (EPI) series.

At the inception of the study, a BOLD EPI series was used during the Emotional Face Categorization/Conflict Task (2 runs of 7 min each) [50, 51]. After enrolling a cohort of 107 patients and 52 healthy participants, we substituted a reward paradigm and an implicit go no-go task. These tasks allow for the assessment of reward networks and attentional biases for affectively laden stimuli [52]. The substitution, rather than addition, of the functional tasks was necessary to maintain a feasible total time for participants in the scanner. The new tasks involve a 12-min hedonic function task [53, 54], where participants receive feedback and earn small monetary rewards while choosing correct responses among sets of visual stimuli and a 10-min affective go/no-go task to a series of stimuli that contain emotional content [55]. Stimulus sizes, instructions to participants, and support materials are standardized across sites. All behavioural data are captured using E-Prime software version 2.0 or higher (Psychology Software Tools, Sharpsburg, PA, USA).

##### Electroencephalography

The fMRI tasks were translated into analogous versions for electroencephalography (EEG), which is carried out at 4 sites, again using several equipment models (Biosemi ActiveTwo [BioSemi, Amsterdam, Netherlands], BrainVision Recorder/QuickAmp [Brain Products, Munich, Germany], Compumedics NeuroScan [Compumedics USA, Charlotte, NC, USA] , EGI Geodesic [Electrical Geodesics, Eugene, OR, USA]) that require cross-site consultation and standardization. All sites used a minimum of 64-channel caps and conductive electrode gels, with 53 common electrodes identified across the different systems. EEGs are digitized continuously (bandpass 0.04–100 Hz with 1000 Hz sampling rate) and all electrodes are referenced to the vertex (Cz) electrode.

At each session, participants complete a 10-min fixation resting state sequence with eyes open, followed by a 10-min resting state sequence with eyes closed. As with fMRI, the Emotional Face Categorization/Conflict Task was used for EEG when the study began, but was later changed to a task querying anhedonia in MDD and an affective go/no-go task, also using E-Prime software. Ocular artifacts, such as blinks, saccades, and lateral movements, are removed by independent component analysis (ICA) as implemented in EEGLAB [56] and performed using MATLAB (MathWorks, Natick, MA, USA).

#### Molecular platform

Blood and urine samples for genomic and proteomic analyses are collected from all participants at Weeks 0, 2, 8 and 16. Following blood draws at Weeks 0, 2, and 8, total RNA is immediately isolated from leukocytes and stabilized using the LeukoLOCK filter apparatus (Thermo Fisher Scientific, Waltham, MA, USA), which also depletes globin mRNA to improve the utility of samples for expression profiling and other applications. For patients with MDD, additional blood samples are collected at Week 4 for pharmacogenetic analysis and at Weeks 2, 10, and 16 for drug levels.

Standard Operating Procedures (SOPs) for sample receipt and accessioning, nucleic acid extraction, quality assessment, data tracking, DNA and RNA preparation for assays, scanning, and feature extraction are in place.

Objectives of the molecular platform are to investigate 1) candidate biomarkers for disease state or drug response in baseline samples in patients and healthy participants, 2) global DNA alterations that may correlate with disease state or drug response using baseline samples, and 3) dynamic molecules in pre- and post- treatment samples to identify pathways of drug response and select additional targets for investigation by targeted methods.

De-identified specimens are transferred to and stored at the Douglas Mental Health University Institute biorepository in accordance with regulatory guidelines and best practices for biobanking. A subset of samples is transferred to other CAN-BIND sites for specific analyses. Material transfer agreements between sites were established at the outset of the study.

Targeted analyses include DNA single-nucleotide polymorphism (SNP) open-array analysis to identify and sequence variants that correlate with disease state or drug response [57], along with studies using established methods for profiling of mRNA and miRNA sequences, histone modifications, and methylation status across the genome [58]. Proteomic analysis by selected-reaction monitoring mass spectrometry (SRM-MS) with state-of-the-art hybrid triple quadrupole/linear ion trap liquid-chromatography mass spectrometry (5500 and 4000 QTRAPs), will be used for relative quantification of high-interest plasma proteins within biological pathways with purported relationships to MDD [59].

Exploratory analyses include RNA-Seq for miRNA from blood and from plasma using either a HiSeq (Illumina, San Diego, CA, USA) or Proton (Thermo Fisher Scientific, Waltham, MA, USA) platform. DNA oxidative damage to guanine will be evaluated by measuring levels of 8-hydroxy-2-deoxyguanosine (8-OhdG) using a competitive ELISA analysis kit (StressMarq BioSciences, Victoria, BC, Canada). Cytosine oxidation will be measured using an ELISA-based assay to assess the levels of 5-hydroxymethylcytosine (MethylFlash™Methylated DNA Quantification Kit; Epigentek Group, Farmingdale, NY, USA). Global DNA methylation (i.e., 5-methylcytosine) will be evaluated using an ELISA-based method (Sigma-Aldrich, Darmstadt, Germany). Inflammatory markers in blood will be measured using standard antibody-based immunoassays.

### Data management

Each site has entered a standardized Participation Agreement with OBI to facilitate transfer of both raw and processed/de-identified data, in accordance with OBI’s Governance Policy and with any specific conditions required by each institution’s local legislative and/or ethical policies.

De-identified electronic data from all sites are aggregated for data analyses using multiple bioinformatics approaches. The OBI’s Centre for Ontario Data Exploration (“Brain-CODE”, https://www.braincode.ca/) is an online neuroinformatics platform that allows researchers to collaborate across distances and work more efficiently, and ultimately, to promote new discoveries to improve patient care. Brain-CODE provides the rare capability of supporting scientific inquiry and analytics across multiple brain diseases and modalities by integrating clinical, imaging, pathology, and genomics data. Standard variable definitions and formats are used so that investigators collect data consistently across studies and modalities. This will reduce variability in data collection and facilitate comparisons across diseases, merging of data sets and meta-analyses.

In storing sensitive patient information, Brain-CODE employs sophisticated security systems and utilizes Privacy by Design (PbD) to protect privacy by embedding it into the design specifications of technologies, business practices, and physical infrastructure [7]. The OBI also works with several research ethics boards and related organizations to streamline the project review and approval process; and develops opportunities to link or integrate Brain-CODE with other provincial, national, or international databases to augment its analytical power. With this unique complement of capabilities, Brain-CODE is the first database of its kind in the world.

Brain-CODE is deployed at the High Performance Computing Virtual Laboratory (HPCVL) data centre at Queen’s University in Kingston, Ontario. The HPCVL is a Compute Canada high-performance computing consortium that supports regulatory-compliant (e.g., ICH E6, 21 CFR Part 11, HIPAA, PIPEDA) processes for securing privacy of healthcare data [60]. All data are collected, processed, maintained, and stored in Canada.

Online data/images can be accessed only on secure websites via restricted portals requiring unique usernames and passwords for each member of the study team. User profiles are assigned only to study personnel requiring access to enter/verify data, and credentials for each user are vetted by the program manager.

Specific data-collection platforms with Brain-CODE include:

1. Brain-CODE Subject Registry, a secure portal used by study centres in Ontario to link encrypted personal health information from study participants to provincial health/administrative databases;
2. OpenClinica Enterprise, a regulatory-compliant, web-based electronic data capture (EDC) system and database for demographic and historical data, diagnostic information, and clinician-rated assessments and scales (OpenClinica, Waltham, MA, USA);
3. LimeSurvey e-PRO Questionnaires, an open-source survey tool used by participants for direct entry of the 20 self-reported measures using a laptop or tablet while attending clinic visits (LimeSurvey, Hamburg, Germany);
4. SPReD (originally the Stroke Patient Recovery Research Database), a comprehensive online repository powered by the open-source Extensible Neuroimaging Archiving Toolkit (XNAT) imaging informatics platform [61]. Structural and functional MRI data are first converted to DICOM (Digital Imaging and Communications in Medicine) format at each site prior to uploading [62], whereas EEG data are uploaded as raw files for subsequent standardization to 58 channels and conversion into a universally readable format in EEGLAB. Supplementary results, such as behavioural and physiological data, and session notes, are uploaded through a special sub-process. All files must be correctly labeled in accordance with SPReD’s organizational structure and naming conventions; an automated pipeline scans for errors on a daily basis; and
5. BASE (BioArray Software Environment) and LabKey, open-source laboratory-information management systems that enable tracking and management of proteomics and genomics workflows, experiments, and raw data [63, 64].

Clinical data support is provided by Indoc Research for OpenClinica and LimeSurvey, and by Rotman Research Institute/Baycrest Health Sciences for SPReD. For clinical and self-rated measures, source data verification is completed using a remote, risk-based monitoring system that includes centralized processes for cleaning and extracting data. Future CAN-BIND projects (see below) will use secure, customizable Research Electronic Data Capture (REDCap) tools for all clinical and self-reported data [65].

MRI data undergoes manual quality control (QC) procedures where trained raters check for visual artifacts and then grade scans as usable or rejected; in the latter case, rescanning is recommended if possible within the study timeline. For fMRI data, preprocessing includes conversion from DICOM to NIfTI (Neuroimaging Informatics Technology Initiative) format [66] prior to identifying the volume with the least motion, to which the remaining volumes are registered.

Converted EEG data are resampled to 512 Hz with channels re-referenced to Cz before digital filters are applied (high pass = 0.5 Hz, low pass = 100 Hz) and trigger codes are standardized.

For a period following study closure, CAN-BIND data are protected by OBI for the exclusive use of the investigative team and its collaborators. However, in the future, de-identified CAN-BIND data may be shared by OBI with other collaborators and third parties for research purposes. These datasets could be made available to other clinical research teams with similar datasets for comparison of treatment outcomes in other psychiatric conditions (such as dementia, schizophrenia, and bipolar disorder). Eligible third parties will be recognized researchers or organizations who have submitted detailed study plans and ethics boards approvals to the OBI. Before OBI discloses the de-identified data to any third party, it will enter into an agreement with the latter to protect confidentiality and ensure correct usage of data.

### Analyses

We will examine several outcomes, including responders at Week 8 who maintained response at Week 16, responders at Week 8 who are non-responders at Week 16, non-responders at Week 8 who respond at Week 16 with augmentation, and non-responders at Weeks 8 and 16, among other outcomes. A reasonably large dataset is required to study adequately the complex clinical, neuroimaging, and molecular factors that contribute to treatment outcomes. Our sample size target for this phase of the CAN-BIND program is 200 patients and 90 healthy participants (290 in total).

To identify standalone features that differ significantly between responders and non-responders, either at baseline or over the course of the study, parametric two-sample t-tests will be used for features with normally distributed data. Otherwise, the non-parametric Mann–Whitney *U* test will be used. A significance threshold of α = 0.05 and multiple testing corrections will be required. Given an input list of up to 25,000 features, of which at least 250 are differentially expressed between responders and non-responders, assuming an overall false discovery rate of 5 %, and applying two-sample t-testing procedures, a minimum of 49 subjects per category are required for power of at least 90 % to correctly identify a given feature which differs significantly between the categories. If nonparametric testing procedures are used, a minimum of 52 subjects per category are required to achieve the same power. Target recruitment for patients with MDD is 200. An estimated response rate of 60 % would yield 120 responders and 80 non-responders. The planned sample size is sufficiently powered for both parametric and non-parametric univariate testing. Multivariate analysis and the development of prognostic signatures of escitalopram response will follow univariate analysis.

Data analyses will be conducted using a suite of commercial and open-source software tools installed on high-end workstations at HPCVL. While data from each analytical platform will be independently analyzed using the methods described above, important relationships (either correlational or causal) between modalities can be detected through an integration of data collected across assessment platforms for a given study subject.

To manage the integrated analyses of this complex data set, we have established a Data Science Advisory Team consisting of the Principal Investigators, domain-specific experts (e.g., molecular or imaging team members), informatics advisors, and operations support. This team will oversee processes for data cleaning, preprocessing, integration, quality control and overall analytics plans for each of the core research activities. Cross-domain as well as domain-specific data interrogation will be conducted by Working Groups responsible for applying specific analytic approaches according to their expertise and contributing to a master Statistical Analysis Plan. Conventional univariate and multivariate statistical approaches will address hypothesis-driven investigations, while a variety of analytics pipelines with both supervised and unsupervised techniques will allow exploratory analyses. Machine-learning approaches, including principle component analysis, random forests, and support vector machines will be used to identify subgroups of patients with shared symptoms and/or biological features that could have clinical relevance.

## Progress to date

Between 2013 and 2015, 159 participants (107 patients and 52 healthy participants) were screened and 134 (85 patients and 49 healthy participants) entered at baseline in the initial protocol with the Emotional Face Categorization/Conflict Task during MRI and EEG sessions, as noted above. Full neuroimaging data (to Week 8) were obtained from 71 patients and 45 healthy participants.

Table 3 lists the clinical characteristics of this first cohort (*N* = 134) that were evaluable at baseline. Independent-samples t-tests showed no significant demographic differences between patients and healthy participants. The patients were moderately depressed at baseline, as reflected by MADRS, QIDS-SR, and CGI-S mean scores, as well as moderately impaired as assessed by psychosocial (SDS) and work (LEAPS) functioning scales.

**Table 3:** Clinical characteristics of the first cohort (*N* = 134)

Characteristic	Patients with MDD (*N* = 85)	Healthy participants (*N* = 49)
Female:Male, *N* (%)	50:35 (59 %:41 %)	32:17 (65 %:35 %)
Age in years, mean (SD), range	36.1 (12.5), 19–61	32.5 (10.2), 20–57
Ethnicity^a^, *N* (%)		
Aboriginal	0	0
Arab	3 (4 %)	1 (2 %)
Asian	9 (11 %)	9 (18 %)
Black	1 (1 %)	0
Latin American/Hispanic	5 (6 %)	2 (4 %)
White	59 (69 %)	35 (71 %)
Other	5 (6 %)	2 (4 %)
Mixed	3 (4 %)	0
Marital status, *N* (%)		
Never Married	48 (57 %)	26 (53 %)
Separated	7 (8 %)	1 (2 %)
Married	16 (19 %)	12 (25 %)
Divorced	7 (8 %)	3 (6 %)
Domestic Partnership	5 (6 %)	6 (12 %)
Widowed	2 (2 %)	1 (2 %)
Occupational status, *N* (%)		
Working now	45 (53 %)	28 (57 %)
Disabled (permanent or temporary)	13 (7 %)	0
Temporary leave	5 (3 %)	0
Looking, unemployed	10 (5 %)	3 (6 %)
Student	8 (4 %)	15 (31 %)
Retired	1 (1 %)	0
If employed, number of hours scheduled to work over the past 2 weeks: mean (SD)	50.0 (27.2)	53.8 (25.8)
If employed, number of hours missed due to symptoms over the past 2 weeks: mean (SD)	10.5 (18.2)	0.1 (0.6)
Education, years: mean (SD)	14.1 (2.0)	15.9 (2.9)
Age of onset of MDD, years:		
mean (SD), range	20.6 (10.7), 5–55	n/a
Single episode:Recurrent, *N* (%)	24:61 (29 %:71 %)	n/a
No. previous episodes, mean (SD)	4.3 (2.8)	n/a
Current episode duration		
≤12 months	39 (46 %)	n/a
1–2 years	13 (15 %)	
>2 years	30 (35 %)	
Unknown/not reported	3 (4 %)	
Median duration (range), months	14.5 (3–151)	
Comorbidities^b,c^		
Substance-Related Disorders	7 (8 %)	n/a
Anxiety Disorders	67 (79 %)	
Eating Disorders	1 (1 %)	
Stable medical conditions	52 (61 %)	
Use of antidepressants during current		
episode, *N* (%)	60 (71 %)	n/a
No. antidepressants used, mean (SD)	1.7 (1.5)	
Baseline MADRS, mean (SD)	29.9 (6.0)	0.4 (1.0)
Baseline YMRS, mean (SD)	2.2 (1.8)	0.3 (0.7)
Baseline CGI Severity, mean (SD)	4.7 (0.8)	1.0 (0.0)
Baseline QIDS-SR, mean (SD)	15.9 (4.3)	2.1 (1.7)
Baseline SDS, mean (SD)	16.6 (7.5)	0.0 (0.0)
Baseline LEAPS, mean (SD)	14.1 (6.1)	1.9 (2.7)

^a^Categories adapted from ethnic-origin groups listed in national census questionnaires [67]

^b^Based on DSM-IV-TR, as determined by the Mini International Neuropsychiatric Interview

^c^Percentages may not add up to 100 % because patients may have more than 1 comorbid condition

*MDD* Major depressive disorder, *SD* Standard deviation, *MADRS* Montgomery Åsberg Depression Rating Scale, *YMRS* Young Mania Rating Scale, *CGI* Clinical Global Impression, *QIDS-SR* Quick Inventory of Depressive Symptomatology, Self-Report, *SDS* Sheehan Disability Scale, *LEAPS* Lam Employment Absence and Productivity Scale

## Discussion

The CAN-BIND study is a unique biomarker-discovery initiative in its mandate, scope, organizational structure, and integrative approach. First, the study collects systematic outcome data in the clinical, neuroimaging, and molecular domains in an agnostic fashion, with no preconceived identification of biomarkers for depression. Second, data are aggregated and integrated using a secure informatics platform that facilitates collaborative data sharing and rigorous quality-control processes across sites. Third, cross-domain and domain-specific analyses are undertaken using high-dimensional mathematical models supplemented with conventional statistical tools.

While CAN-BIND-1 focuses on pharmacotherapy, subsequent protocols will address biomarkers in treatment response using other evidence-based interventions for MDD, including transcranial magnetic stimulation, cognitive behaviour therapy, cognitive remediation, and others. Additional studies using CAN-BIND platforms to examine integrated biomarkers involve youth at risk for serious mental illness, suicide risk, and effects of childhood maltreatment on stress sensitivity and reward responsivity. CAN-BIND also operates a dedicated reverse-translation platform with various pre-clinical groups that investigate neuropharmacology of hedonic function in laboratory rats, zebrafish high-throughput screening, electrophysiological and behavioural impact of medications, and microRNA/inflammatory markers. CAN-BIND activities are broadly disseminated through multimodal, iterative knowledge translation/knowledge exchange initiatives that include ongoing collaboration with patients, family members, and other stakeholders in the community. Over time, CAN-BIND will expand with new collaborations with academic health centres, industry partners, and mental health networks.

## Conclusion

CAN-BIND is a large Canadian collaborative research endeavor that is attempting to discover integrated clinical, imaging and molecular biomarkers of treatment response in MDD. It may also identify clinically relevant subtypes of depression and further our knowledge of the pathogenesis and pathophysiology of MDD. Given the multifaceted study design, we also expect to find novel psychobiological insights that will lead to the generation of new hypotheses to be validated in future studies.

### Ethics approval and consent to participate

Research described in this article has been ethics approved at each participating clinical centre. The ethics committees include: University of British Columbia Clinical Research Ethics Board (Vancouver); University of Calgary Conjoint Health Research Ethics Board (Calgary); University Health Network Research Ethics Board (Toronto); Centre for Addiction and Mental Health Research Ethics Board (Toronto); Hamilton Integrated Research Ethics Board (Hamilton); Queen’s University Health Sciences and Affiliated Teaching Hospitals Research Ethics Board (Kingston). Participants provided written, informed consent for all study procedures.

### Consent for publication

Not applicable - this manuscript does not contain any individual persons’ data.

## Abbreviations

21 CFR Part 11: title 21 of the code of federal regulations [U.S.]; electronic records; electronic signatures, part 11
ATHF: antidepressant treatment history form
BASE: bioArray software environment
BIS/BAS: behavioural inhibition system/behavioural activation system
BOLD: blood oxygenation level-dependent
BPI-SF: brief pain inventory
Brain-CODE: Ontario brain institute centre for Ontario data exploration
BRIAN: biological rhythm interview of assessment in neuropsychiatry
CAN-BIND: Canadian biomarker integration network in depression
CANMAT: Canadian network for mood and anxiety treatments
CBT: cognitive-behavioural therapy
CECA: childhood experience of care and abuse
CGI: clinical global impression
CNS VS: central nervous system vital signs
Cz: vertex [in EEG]
DARS: dimensional anhedonia rating scale
DICOM: digital imaging and communications in medicine
DID: depression inventory development
DNA: deoxyribonucleic acid
DSM-5: diagnostic and statistical manual of mental disorders, fifth edition
DSM-IV-TR: diagnostic and statistical manual of mental disorders, fourth edition, text revision
DTI: diffusion-tensor imaging
ECR-R: experiences in close relationships
EEG: electroencephalography
ELISA: enzyme-linked immunosorbent assay
EPI: echo planar imaging
e-PRO: electronic version of patient-reported outcome
fMRI: functional magnetic resonance imaging
FOV: field of view
GAD: generalized anxiety disorder
GE: general electric
HCL-32: hypomania check-list, 32-item version
HIPAA: health insurance portability and accountability act [U.S.]
HPCVL: high performance computing virtual laboratory
Hz: hertz
ICH E6: international conference on harmonisation of technical requirements for registration of pharmaceuticals for human use, guidance on efficacy, 6 (good clinical practice)
IPAQ: international physical activity questionnaire
iTBS: intermittent theta-burst stimulation
KT/KE: knowledge transfer/knowledge exchange
LEAPS: lam employment absence and productivity scale
LEDS: life events and difficulties schedule
LTE: list of threatening experiences
MADRS: montgomery-asberg depression rating scale
MDD: major depressive disorder
MDE: major depressive episode
MINI: mini international neuropsychiatric interview
miRNA: microRNA
mm: millimetres
MRI: magnetic resonance imaging
mRNA: messenger RNA
ms: milliseconds
NART: national adult reading test
NEO-FFI: NEO five-factor inventory
NIfTI: neuroimaging informatics technology initiative
OBI: Ontario brain institute
QC: quality control
QIDS-SR: quick inventory of depressive symptomatology, self-report
Q-LES-Q: quality of life, enjoyment and satisfaction questionnaire
QTRAP: quadruple/linear ion trap
PbD: privacy by design
PIPEDA: personal information protection and electronic documents act
PRN: *pro re nata* [i.e., in medicine, “as needed”]
PSQI: Pittsburgh sleep quality index
REDCap: research electronic data capture
RNA: ribonucleic acid
rTMS: repetitive transcranial magnetic stimulation
SDS: sheehan disability scale
SexFX: sexual side effects questionnaire
SHAPS: snaith-hamilton pleasure scale
SIGMA: structured interview guide for the MADRS
SMI: serious mental illness
SNP: single-nucleotide polymorphism
SOP: standard operating procedures
SPAQ: seasonal pattern assessment questionnaire
SPReD: stroke patient recovery research database
SRM-MS: selected-reaction monitoring mass spectrometry
TE: echo time [in MRI]
TR: repetition time [in MRI]
TSES: Toronto side effects scale
WHOQoL-BREF: world health organization quality of life assessment
XNAT: extensible neuroimaging archiving toolkit
YMRS: young mania rating scale
